# Defining A Liquid Biopsy Profile of Circulating Tumor Cells and Oncosomes in Metastatic Colorectal Cancer for Clinical Utility

**DOI:** 10.3390/cancers14194891

**Published:** 2022-10-06

**Authors:** Sachin Narayan, George Courcoubetis, Jeremy Mason, Amin Naghdloo, Drahomír Kolenčík, Scott D. Patterson, Peter Kuhn, Stephanie N. Shishido

**Affiliations:** 1 Michelson Center for Convergent Bioscience, Convergent Science Institute in Cancer, Dornsife College of Letters, Arts and Sciences, University of Southern California, Los Angeles, CA 90089, USA; 2 Catherine & Joseph Aresty Department of Urology, Institute of Urology, Keck School of Medicine, University of Southern California, Los Angeles, CA 90033, USA; 3 Norris Comprehensive Cancer Center, Keck School of Medicine, University of Southern California, Los Angeles, CA 90033, USA; 4 Biomedical Center, Faculty of Medicine in Pilsen, Charles University, 32300 Pilsen, Czech Republic; 5 Gilead Sciences, Inc., Lakeside Drive, Foster City, CA 94404, USA; 6 Department of Biomedical Engineering, Viterbi School of Engineering, University of Southern California, Los Angeles, CA 90089, USA; 7 Department of Biological Sciences, Dornsife College of Letters, Arts, and Sciences, University of Southern California, Los Angeles, CA 90089, USA; 8 Department of Aerospace and Mechanical Engineering, Viterbi School of Engineering, University of Southern California, Los Angeles, CA 90089, USA

**Keywords:** liquid biopsy, rare cell, circulating tumor cells, oncosomes, colorectal cancer, heterogeneity, multi-assay, high-definition single-cell assay

## Abstract

**Simple Summary:**

Metastatic colorectal cancer (mCRC) is typified by its tumor heterogeneity and changing disease states, suggesting that personalized medicine approaches could be vital to improving clinical practice. As a minimally invasive approach, the liquid biopsy has the potential to be a powerful longitudinal prognostic tool. We investigated mCRC patients’ peripheral blood samples using an enrichment-free single-cell approach to capture the broader rare-event population beyond the conventionally detected epithelial-derived circulating tumor cell (CTC). Our analysis reveals a heterogenous profile of CTCs and oncosomes not commonly found in normal donor samples. We identified select rare cell types based on their distinct immunofluorescence expression and morphology across multiple assays. Lastly, we highlight correlations between enumerations of the blood-based analytes and progression-free survival. This study clinically validates an unbiased rare-event approach in the liquid biopsy, motivating future studies to further investigate these analytes for their prognostic potential.

**Abstract:**

Metastatic colorectal cancer (mCRC) is characterized by its extensive disease heterogeneity, suggesting that individualized analysis could be vital to improving patient outcomes. As a minimally invasive approach, the liquid biopsy has the potential to longitudinally monitor heterogeneous analytes. Current platforms primarily utilize enrichment-based approaches for epithelial-derived circulating tumor cells (CTC), but this subtype is infrequent in the peripheral blood (PB) of mCRC patients, leading to the liquid biopsy’s relative disuse in this cancer type. In this study, we evaluated 18 PB samples from 10 mCRC patients using the unbiased high-definition single-cell assay (HDSCA). We first employed a rare-event (Landscape) immunofluorescence (IF) protocol, which captured a heterogenous CTC and oncosome population, the likes of which was not observed across 50 normal donor (ND) samples. Subsequent analysis was conducted using a colorectal-targeted IF protocol to assess the frequency of CDX2-expressing CTCs and oncosomes. A multi-assay clustering analysis isolated morphologically distinct subtypes across the two IF stains, demonstrating the value of applying an unbiased single-cell approach to multiple assays in tandem. Rare-event enumerations at a single timepoint and the variation of these events over time correlated with progression-free survival. This study supports the clinical utility of an unbiased approach to interrogating the liquid biopsy in mCRC, representing the heterogeneity within the CTC classification and warranting the further molecular characterization of the rare-event analytes with clinical promise.

## 1. Introduction

Colorectal cancer (CRC) is the world’s third most common cancer and second leading cause of oncology-related deaths [1]. Most notably, CRC solid tumors are marked by their extensive cellular heterogeneity and proliferation owed to the rapid rate of epithelial self-renewal in the intestines [2,3,4]. The variety of tumor microenvironments, genetic mutations, and disease subtypes suggests that real-time individualized analysis and subsequent clinical decision-making could improve patient outcomes [5,6,7].

As a minimally invasive procedure, the liquid biopsy has the potential to be that critical element in the longitudinal evaluation of CRC by characterizing the disease’s pathophysiology and mechanisms of metastasis [8,9,10]. Much of the current liquid biopsy analysis focuses on two primary biomarkers: circulating tumor cells (CTCs) and cell-free DNA (cfDNA). Additionally found in the blood, carcinoembryonic antigen (CEA) is one of the foremost prognostic hallmarks of CRC [11,12]. Previous investigations have correlated CTC counts to CEA levels, with the two used in conjunction to accurately predict survival outcomes [13]. Generally, a higher number of CTCs indicates poorer patient outcomes in CRC [14,15], although nuances emerge when considering morphologically defined CTC subtypes and the change in cell populations over time [16,17]. With the variety of detectable biomarkers in circulation, the liquid biopsy could aid in tackling some of the clinical challenges of CRC.

Previously, CellSearch^®^ (Menarini, Raritan, NJ, USA) was the first platform to receive regulatory approval in CRC via a 510(k) clearance for the enumeration of CTCs to monitor metastatic colorectal, breast and prostate cancer [18]. The platform uses an enrichment-based methodology for the detection of a singular type of CTC defined by the expression of epithelial cell adhesion molecule (EPCAM) and cytokeratin (CK) without the expression of CD45 [19], thereby limiting the liquid biopsy field’s current understanding of CTC. CellSearch^®^ uses a uniform threshold for CTC positivity at any timepoint during the patient’s treatment. In CRC, this threshold is 3 CTCs/7.5 mL of blood [20]. CellSearch^®^ has shown that a higher frequency of CTCs is associated with poorer overall survival (OS) and progression-free survival (PFS) in CRC [21,22]. However, CellSearch^®^ and similar systems are not commonly utilized by clinicians treating CRC [23,24]. This limited utility could be attributed to a lack of timepoint-specific standards [18] and infrequent CTC kinetics analysis, despite its clinical promise [16]. Most early-generation liquid-biopsy platforms employ enrichment-based approaches that detect a limited CTC population in the peripheral blood (PB) of CRC patients [25,26,27] and overlook other cellular subtypes with nuanced survival implications [16,28]. In a cancer type known for its tumor heterogeneity like CRC, enrichment-based approaches limit the liquid biopsy’s potential clinical utility, thereby warranting an unbiased single-cell approach that focuses on all rarity in the bloodstream.

This study utilized the third-generation high-definition single-cell assay (HDSCA3.0), which is a validated “no cell left behind” immunofluorescence (IF) assay that detects and characterizes all rare events from the liquid biopsy [25,28,29,30,31,32,33]. Commercialized by Epic Sciences, it has demonstrated clinical utility as a predictive marker in prostate cancer [34,35,36]. Furthermore, it allows for downstream genomic and proteomic analysis [37,38] and adheres to the standards of the Blood Profiling Atlas Commons [39]. A prior investigation into a cohort of metastatic CRC (mCRC) patients with HDSCA2.0 revealed a 35% CTC positivity rate [25], comparable to CellSearch^®^ positivity rates among similar cohorts [30,37,40]. A subsequent study with the same platform highlighted the importance of CTC subtypes, time of sample collection and changes in cellular populations during treatment in understanding the value of the liquid biopsy in mCRC patient care [16]. Beyond CTC subtypes, prior studies have identified other rare cells such as circulating endothelial cells (CECs) [41,42] with prognostic implications in mCRC. In addition to rare cellular analytes, tumor-derived oncosomes and extracellular vesicles have been shown to promote tumorigenesis and chromosomal deletion across cancer types [43,44,45]. Now, the third generation of HDSCA detects a heterogeneous CTC and oncosome population with various surface biomarkers and an unbiased computational methodology for the detection of epithelial, mesenchymal, endothelial and immune cells [46,47]. Herein, we analyzed 18 PB samples from 10 mCRC patients using two IF protocols to represent a comprehensive CTC and oncosome liquid-biopsy profile, highlighting previously unidentified rare events and correlating analytes to patient outcomes.

## 2. Materials and Methods

### 2.1. Study Design

This study includes a total of 18 PB samples collected between May 2016 to March 2017 from 10 patients with mCRC. Patients were found as part of the GS-US-296-0101 phase I clinical trial (#NCT01803282) evaluating the safety and tolerability of a novel therapeutic in combination with standard-of-care chemotherapy in two different mCRC indications. Apart from their diagnosis of CRC and survival data, no other clinical or demographic information was available for this study cohort per IRB protocol at the time of enrollment. Patients 1, 3, 5, 6 and 9 were first-line inoperable mCRC patients receiving the test compound in combination with mFOLFOX6 and bevacizumab. Patients 2, 4, 7, 8 and 10 were second-line inoperable patients receiving the test compound in combination with FOLFIRI and bevacizumab. PB samples were collected on cycle 1, day 1 and cycle 3, day 1 of therapy, referred to as Draw 1 and Draw 2, respectively. All patients progressed, and PFS was provided for 9 of the 10 patients. In addition, PB samples from 50 normal donors (ND) with no known pathology were collected and provided by Epic Sciences (San Diego, CA, USA).

### 2.2. Blood Processing

PB samples were collected in 10 mL collection tubes (Cell-free DNA, Streck, La Vista, NE, USA) and were processed as previously described [30,47]. As a brief synopsis, after red blood cell lysis, the nucleated cell fraction was plated as a monolayer of ~3 million cells per slide (Marienfeld, Lauda, Germany) before cryobanking at −80 °C.

### 2.3. IF Staining Protocols

Samples were analyzed with the previously described workflow for high-resolution imaging and the characterization of tumor cells at a single-cell level [30]. Slides were stained by the IntelliPATH FLX^TM^ autostainer (Biocare Medical LLC, Irvine, CA, USA) in batches of 50. Our validated HDSCA protocols utilize a cocktail of pan-cytokeratin (CK), CD45 antibodies and DAPI [30,31]. In further detail, samples were fixed with a 2% neutral buffered formalin solution (VWR) for 20 min followed by permeabilization using 100% cold methanol for 5 min and blocking nonspecific binding sites with 10% goat serum (Millipore) for 20 min. This is followed by an antibody cocktail consisting of mouse IgG1/Ig2a anti-human cytokeratins 1, 4, 5, 6, 8, 10, 13, 18 and 19 (clones: C-11, PCK-26, CY-90, KS-1A3, M20, A53-B/A2, C2562, Sigma, St. Louis, MO, USA), mouse IgG1 anti-human cytokeratin 19 (clone: RCK108, GA61561-2, Dako, Carpinteria, CA, USA) and mouse anti-human CD45:Alexa Fluor^®^ 647 (clone: F10-89-4, MCA87A647, AbD Serotec, Raleigh, NC, USA). To complete the staining, slides were incubated with Alexa Fluor^®^ 555 goat anti-mouse IgG1 antibody (A21127, Invitrogen, Carlsbad, CA, USA) and 4′,6-diamidino-2-phenylindole (DAPI; D1306, ThermoFisher) prior to being mounted with a glycerol-based aqueous mounting media [48].

Two distinct IF protocols were applied: 1) Landscape and 2) CDX2-targeted. The Landscape protocol identifies epithelial, mesenchymal, endothelial and immune cells through the addition of 100 ug/mL of a goat anti-mouse IgG monoclonal Fab fragments (115-007-003, Jackson ImmunoResearch, West Grove, PA, USA), rabbit IgG anti-human vimentin (Vim) (clone: D21H3, 9854BC, Cell Signaling, Danvers, MA, USA) as a fourth color and mouse IgG1 anti-human CD31:Alexa Fluor^®^ 647 mAb (clone: WM59, MCA1738A647, BioRad, Hercules, CA, USA) to the CD45 channel, hereafter referred to as CD45/CD31 [47]. The CDX2-targeted protocol utilizes the colon-specific CDX2 monoclonal antibody EPR2764Y (Abcam, Cambridge, UK) as a fourth color for further characterization [25]. CDX2 is a transcription factor expressed throughout the intestinal epithelium and has been effectively used as a marker for intestinal carcinomas [49]. The ND samples were stained with the Landscape protocol.

### 2.4. Scanning and Analysis

Slides were imaged at 2304 frames per slide using automated high-throughput fluorescence scanning microscopy at 100× magnification with exposures and gain set to yield the same background intensity level for normalization purposes. The numeration of cell classifications was converted to concentration based on the sample leukocyte concentration measured at processing and the number of DAPI-positive nuclei detected. White blood cell (WBC) counts in the PB sample were determined automatically (Medonic M-series Hematology Analyzer, Clinical Diagnostic Solutions Inc., Fort Lauderdale, FL, USA). The number of WBCs per slide was utilized in the calculation of the exact amount of blood analyzed, leading to rare-event enumerations presented in events/mL. Cells of interest were further imaged at higher magnification (400×). IF signal expression is categorized as filamentous, diffuse or punctate, as previously described [50].

### 2.5. Rare Event Detection Approach

Cells were identified via a rare-event detection method termed OCULAR [47,48]. This algorithm uses feature extraction, principal component analysis (PCA) and hierarchical clustering on the principal components to achieve four distinct tasks: fluorescent image feature extraction (761 parameters), rare-event detection (distinguishing between common and rare DAPI-positive events and DAPI-negative events), rare-cell classification and report generation. Image analysis was performed as previously reported [47]. In brief, all events were segmented to generate nuclear and/or cytoplasm masks for feature extraction, which was followed by a dimensionality reduction using principal components and hierarchical clustering to separate common cells (mainly WBCs) and rare cells in each image frame. The manual classification of rare events into CTC or oncosome subgroups was conducted based on biomarker expression in the four fluorescence channels for each IF protocol. Classifications were validated by multiple hematopathologist-trained technical analysts. Previously described as large extracellular vesicles in the context of HDSCA [46,48,51], oncosomes were identified as circular, DAPI-negative events with positive CK expression [44,52,53]. Initially presented as adjacent to nucleated common cells or as individual DAPI-negative events, oncosomes were manually classified and confirmed by trained analysts. The nomenclature for channel-type classifications utilizes those positive channels (Landscape example: CK|Vim = DAPI-positive, CK-positive, Vim-positive, CD45/CD31-negative). Oncosome channel-type classifications are preceded by the abbreviation “Onc” (Landscape example: Onc CK|Vim = DAPI-negative, CK-positive, Vim-positive, CD45/CD31-negative). While rare cell types are predominantly referred to by their channel-type classification, two specific CTC populations, epithelial CTCs (Epi.CTC) and mesenchymal CTCs (Mes.CTC), are evaluated in this study. As previously described [48], Epi.CTCs are CK-positive, Vim-negative and CD45/CD31-negative with a clearly defined nucleus (DAPI). Mes.CTCs are CK-positive, Vim-positive and CD45/CD31-negative and have clearly defined nuclei.

### 2.6. Multi-Assay Analysis

With sample-matched slides stained by both IF protocols (Landscape and CDX2-targeted), OCULAR’s uniform examination of the rare events allowed for a multi-assay analysis. OCULAR derives 761 morphological parameters from IF 100× magnification images of detected cellular events. To perform the multi-assay analysis, we selected a subset of 8 morphometric parameters, consisting of the most representative features. With both IF protocols containing DAPI and CK, the median intensity for these two channels were chosen. In addition, we included the eccentricity and area of both the cell and nucleus, the ratio of nuclear to cellular area and the average distance of the cell outline to the center of the nucleus. Using these 8 shared morphometric features, 5661 rare cells across both assays were grouped together using a hierarchical clustering model. An agglomerative clustering algorithm was used, imported from the scikit-learn library version 0.23.2 [54] in Python. We used a Euclidian metric to compute the distance and the ward linkage criterion. In addition, 111 cells and the oncosome population were removed after manual inspection from the clustering due to highly aberrant nuclear and membrane masking. The cohort’s rare-cell population with various cluster assortment options are included as part of an interactive webpage that can be used for additional analyses and discovery (https://pivot.usc.edu/pivot/CRC_MultiAssay.html). The optimal number of clusters from 2 to 16 was selected based on quantitative cluster-separation metrics and the separation of noteworthy, rare cell types. Based on the silhouette average method, 8 clusters provided the optimal separation between the cell groups. When increasing the number of clusters from 8 through 12, cluster 1 is the primary group undergoing rearrangement, but subsequent cluster combinations do not perform as well in the silhouette average metric or in their division of rare cell types of interest. Alongside the clustering approach, a Spearman’s rank correlation [55] analysis was performed between the rare-event enumerations of the two IF protocols.

### 2.7. Survival Analysis

To perform survival analysis on the 9 patients with known progression time, the Spearman’s rank correlation coefficient [55] was calculated for all liquid-biopsy analytes versus the PFS. Then, only liquid-biopsy analytes with statistically significant entries were used for subsequent visualization. To visualize the statistically significant entries, the Kaplan–Meier (KM) curves [56] for the patients’ PFS were plotted. Then, to depict the effect of a statistically significant liquid-biopsy analyte on PFS, the patients were stratified based on their respective counts per milliliter of blood for the given liquid-biopsy analyte. The stratification was done by using the median counts per milliliter of blood as a threshold, separating the patients into two groups. Finally, the two patient subgroups were plotted together with the original KM curve of the population. The statistical analysis was done using scipy [57], and the KM curves were plotted using scikit-survival library [58] in Python.

### 2.8. Statistical Analysis

The distinction between the rare-event enumerations of the mCRC and ND samples was determined by a Wilcoxon rank sum test [59]. Statistical correlations between rare-event enumerations were performed using the Spearman’s rank correlation coefficient [55]. A correlation was significant if the two-tailed *p*-value ≤ 0.05. The statistical analysis was done using scipy [57] in Python.

## 3. Results

### 3.1. Landscape Rare-Event Detection: Rare Cells and Oncosomes

**Figure 1:**
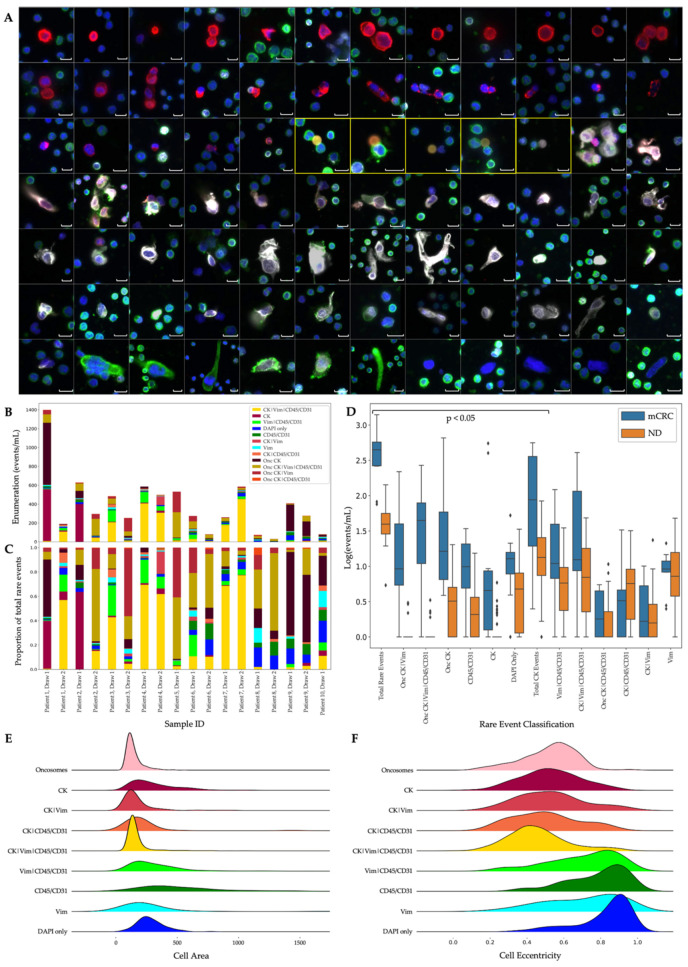
Landscape-stained samples analyzed by OCULAR. (**A**) Representative gallery from the metastatic colorectal cancer (mCRC) cohort showing the morphological heterogeneity of the detected rare events. DAPI: blue, cytokeratin (CK): red, Vim: white, CD45/CD31: green. Events from each of the 10 patients are represented in the gallery. The five oncosomes displayed are bordered by yellow boxes. Events are ordered by decreasing CK signal intensity. Images taken at 400× magnification. Scale bars represent 10 µm. (**B**) Rare-event enumeration (events/mL) and (**C**) frequency (%) per patient and draw. (**D**) Enumeration comparison of Draw 1 mCRC and normal donor samples ordered by statistical significance. Symbols indicate outliers. The first 8 classifications from the left are different between mCRC and normal donors (*p* < 0.05). (**E**) Cellular area and (**F**) cellular eccentricity per rare-event classification detected in the mCRC cohort.

PB samples were stained with the Landscape IF protocol and analyzed by OCULAR to identify the rare events with biomarkers highlighting epithelial, endothelial, mesenchymal and immune cell origin. Rare-event frequencies, enumerations and sample positivity (≥5 events/mL) from the Landscape and CDX2 IF protocols are reported in Table 1. For the 18 mCRC samples analyzed, on average 0.53 (standard error 0.05, median 0.53, range 0.22–0.97) mL of PB was used for 1 test, thus the sensitivity of the analysis is limited by the blood volume characterized. A representative subset of the rare-cell and oncosome populations detected in the Landscape-stained samples is displayed in Figure 1A, with enumerations, frequencies and comparisons to the ND cohort, and select morphometrics provided in Figure 1B, 1C and 1D, respectively. The sample enumeration of the ND cohort is depicted in Supplemental Figure S1.

For all mCRC samples, total rare-event (total cells and oncosomes) detection had a median of 287.46 (mean 387.74 ± 75.33) events/mL. For ND samples, total rare-event detection had a median of 40.05 (mean 49.96 ± 4.18) events/mL. A significant difference was observed between the mCRC patients and ND (*p* < 0.0001; Figure 1D).

Rare cells comprised 58.25% of the total rare-event profile from the Landscape-stained mCRC samples. The rare cells detected in mCRC patient samples were highly heterogenous in their signal expression and morphology (Figure 1E,F). Total rare-cell detection for the mCRC samples had a median of 124.66 (mean 224.80 ± 51.55) cells/mL. The ND samples presented with a median rare-cell detection of 34.46 (mean 43.21 ± 3.94) cells/mL. A significant difference in total rare-cell detection was observed between the mCRC patients and ND samples (*p* = 0.0112).

Total CK-positive events were detected with a median of 45.41 (mean 169.66 ± 46.65) events/mL from all mCRC samples. The ND samples had a median of 12.39 (mean 18.96 ± 2.70) CK-positive events/mL. There was a statistically significant difference in total CK-positive event detection between the mCRC patients and ND samples (*p* = 0.0070). The total CK-positive cell population constituted 75.47% of all the rare cells. Epi.CTCs were detected with a median of 3.55 (mean 57.00 ± 36.36) cells/mL from mCRC patient samples, which is a significantly higher incidence compared to ND samples (*p* = 0.0023). Epi.CTCs were only identified in 7 of 18 mCRC samples, with 2 samples containing 92.58% of the total cohort’s population. This speaks to the limited frequency of CK-only CTCs in the liquid biopsy of mCRC, here using approximately 0.5 mL of PB. Similarly, Mes.CTCs were only found in 6 of 18 samples and with a median of 1.40 (mean 7.60 ± 1.40) cells/mL in the mCRC cohort. There was not a significant difference in the Mes.CTC count between the mCRC and ND samples. The CK|Vim|CD45/CD31 cells were the most frequent rare cell across the mCRC cohort, but no statistically significance difference was detected between the mCRC and ND samples.

Other detectable rare cells in the mCRC samples included morphologically distinct CD45/CD31-only (median 8.05; mean 10.29 ± 2.44), DAPI-only (median 10.84; mean 13.69 ± 2.90), Vim-only (median 7.30; mean 6.64 ± 1.27) and Vim|CD45/CD31 (median 7.79, mean 24.52 ± 8.73) cells/mL. The DAPI-only cells were detected at a higher prevalence in mCRC samples compared to the ND samples (*p* = 0.0047).

**Figure 2:**
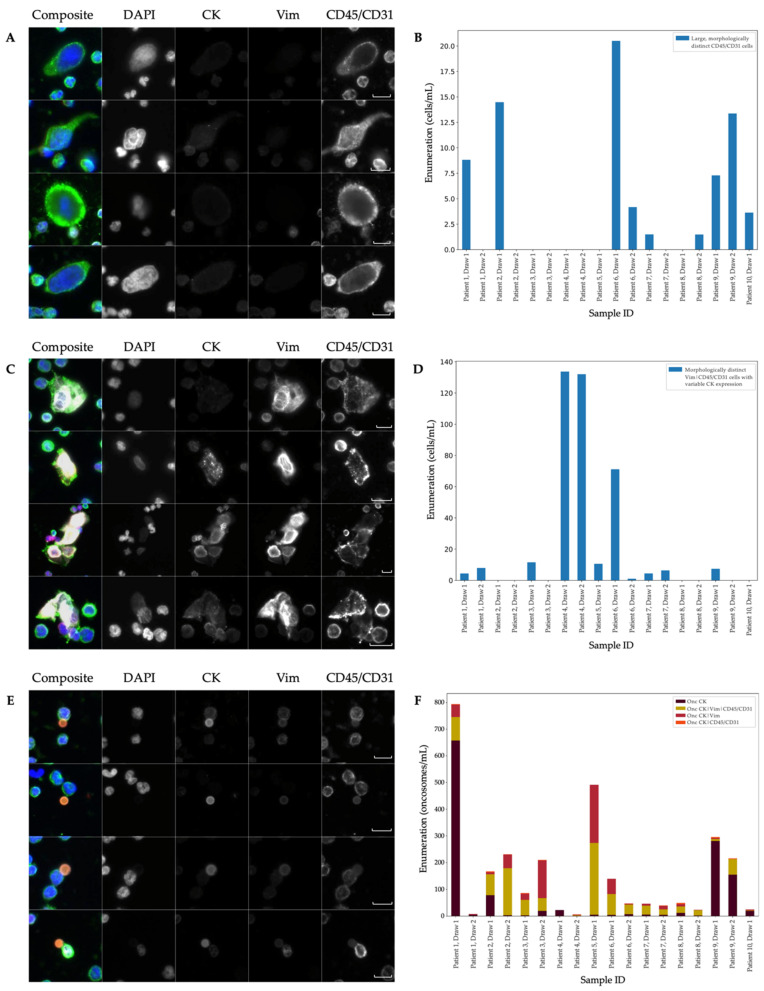
Select rare-event populations in the Landscape-stained metastatic colorectal cancer (mCRC) samples. (**A**) Panel gallery images of the large, morphologically distinct CD45/CD31 cells. (**B**) Large, morphologically distinct CD45/CD31 cell enumeration per patient and draw. (**C**) Panel gallery images of the morphologically distinct Vim|CD45/CD31 cells with variable cytokeratin (CK) expression. (**D**) Morphologically distinct Vim|CD45/CD31 cells with variable CK expression enumeration per patient and draw. (**E**) Panel gallery images of the oncosome population with differential signal expression (**F**) Oncosome enumeration by channel classifications per patient and draw. Images are taken at 400× magnification. Scale bars represent 10 µm.

Two morphologically distinct cell types were identified as unique subsets of their broader channel-type classifications. The first was the large, morphologically distinct CD45/CD31 cell population shown in Figure 2A. As a subset of the CD45/CD31 channel-type classification, this cell type was found in 9 of the cohort’s 18 samples (Figure 2B). Their distinction highlights the importance of morphological analysis that goes beyond channel-type classifications. The cells of interest possess a punctate CD45/CD31 signal, which is distinct from the diffuse CD45/CD31 signal that typifies the surrounding WBCs. Notable morphometrics quantitatively distinguish this cell group from common WBCs with a larger cell size, larger nuclear size, and higher cellular eccentricity. Even within this category, there is heterogeneity, evidenced by the varying shapes and the nuclear-to-membrane ratios of the cells. These cells are hypothesized to be megakaryocytes.

The second rare-cell group of interest consists of morphologically distinct Vim|CD45/CD31 cells with variable CK expression, as shown in Figure 2C. These cells are found in 11 of 18 samples and across both draws (Figure 2D). The samples from Patient 4 contain the vast majority (81.37%) of the morphologically distinct Vim|CD45/CD31 cells detected in this cohort. The image analysis of these cells revealed a filamentous Vim signal along with a punctate CD45/CD31 signal. The variable CK expression in signal intensity and appearance suggests that there are additional subtypes within this cell type. A total of 45.60% of the total morphologically distinct Vim|CD45/CD31 cell population is CK-positive. Further observation highlights heterogeneous CK expression within the CK-positive subtype, epitomized by the punctate and filamentous signal on the second and third cell of Figure 2C respectively. Beyond the analysis of the IF signal, 35.95% of the morphologically distinct Vim|CD45/CD31 cells found in this cohort are found clustered near one another (bottom of Figure 2C). Additional key morphometrics that distinguish this cell population from surrounding WBCs include a large cell size and eccentric cellular membrane. These cells are hypothesized to be endothelial cells.

In addition to the rare-cell groups, OCULAR identified a sizeable population of oncosomes. Oncosomes accounted for 41.75% of the rare events in the Landscape-stained samples. Morphologically, these vesicles ranged up to the size of neighboring WBCs (~10 µm) and were present in the cellular fraction of blood after centrifugation. As Figure 2E depicts, these vesicles were found in contact with adjacent nucleated cells and in isolation, with 51.09% of this cohort’s oncosome population belonging to the latter. Furthermore, their IF signal was diffuse, suggesting an evenly distributed expression across the vesicle. The oncosomes expressing CK were generally the most prevalent (Figure 2F). As the most common subtype, all 18 samples were positive for Onc CK with a median of 6.64 (mean 71.59 ± 38.39) events/mL. The Onc CK|Vim|CD45/CD31 was positive in 16 of 18 samples and had a median of 34.32 (mean 55.37 ± 16.22) events/mL. The Onc CK|Vim was also present in 16 of 18 samples, with a median of 7.38 (mean 32.72 ± 13.64) events/mL. The Onc CK|Vim|CD45/CD31 and Onc CK|Vim counts were found to be highly positively correlated (*p* = 0.002, τ = 0.68).

In comparison to the mCRC and ND samples, 6 specific channel-type rare-event classifications were statistically distinct across the cohorts (Figure 1D). Three of the significantly different channel-type classifications were rare cells that were detected at a higher prevalence in mCRC samples compared to the ND: Epi.CTC (*p* = 0.0023), DAPI only (*p* = 0.0494) and CD45/CD31 (*p* = 0.0004). Three oncosome channel-type classifications were observed at greater numbers in the mCRC patient samples compared to the ND samples: Onc CK (*p* = 0.0001), Onc CK|Vim (*p* < 0.0001) and Onc CK|Vim|CD45/CD31 (*p* < 0.0001).

### 3.2. Analysis of the CDX2-Targeted Protocol

**Figure 3:**
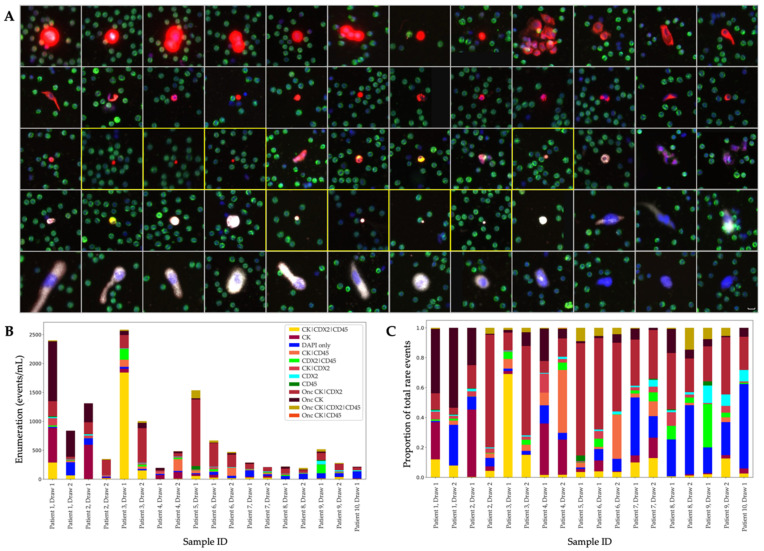
CDX2-targeted samples analyzed by OCULAR. (**A**) Representative rare-event gallery. DAPI: blue, cytokeratin (CK): red, CDX2: white, CD45: green. Events from each of the 10 patients are represented in the gallery. The eight oncosomes displayed are bordered by yellow boxes. Events are ordered by decreasing CK intensity. Images taken at 100× magnification. Scale bar for all images is shown in the bottom right cell, representing 10 µm. (**B**) Enumeration (events/mL) of each channel classification per patient and draw. (**C**) Frequency (%) of each channel classification per patient and draw.

To complement the vast heterogeneity of rare events across the epithelial, mesenchymal and endothelial cell types presented in the Landscape protocol, the CDX2-targeted protocol was utilized to specifically interrogate and identify circulating rare events of colorectal origin. Slides from the same PB tubes were stained with the CDX2-targeted protocol and analyzed by OCULAR, allowing for a sample-matched study design across two IF assays. All rare-event frequencies, enumerations and sample positivity (≥5 events/mL) from the CDX2-targeted protocol are reported in Table 1. A representative subset of the rare-cell and oncosome populations are displayed in Figure 3A, with enumerations and frequencies of the channel-type classifications in Figure 3B,C. Further analysis of the CDX2-targeted cohort by HDSCA’s first generation CK-focused approach is depicted in Supplemental Table S1, Figures S2 and S3.

Rare cells comprised 52.10% of the total rare-event profile from the CDX2-targeted protocol. From the OCULAR analysis, the total CK-positive cell population across all 18 samples had a median of 108.74 (mean 288.43 ± 119.17) cells/mL. The image analysis of the CK-positive cell groups revealed extensive heterogeneity between and within the channel-type classifications. The consistency of the IF signal across the cells of interest varies within categories. Morphological differences were also observed between cells of the same channel type.

With the added CDX2 marker, we were able to evaluate CTCs for their potential colorectal origin. CDX2-positive CTCs or CDX2.CTCs (also referred to as CK|CDX2 in Table 1) were found in 14 of 18 samples, but not in high frequencies, only constituting 2.52% of the total rare-event profile. The first three images from the top left of Figure 3A are representative of this cell type, and, as the gallery shows, these CDX2.CTCs were also found clustered together.

Beyond the CK-positive populations, additional rare cell types were prominent across the CDX2-stained samples: DAPI-only, CDX2-only, CD45-only and CDX2|CD45 classifications (Table 1). The CK-negative rare-cell population detected by OCULAR was positive in all 18 samples, with a median of 87.13 (mean 109.98 ± 18.58) cells/mL. Among this group, a subset of large and eccentric cells with punctate CDX2 expression and sizeable nuclei (see the bottom row of Figure 3A) were found in 11 of the 18 samples. Lastly, all 18 of samples were positive for DAPI-only rare cells, with a median of 45.29 (mean 63.72 ± 12.80) cells/mL using the CDX2-targeted protocol.

### 3.3. Multi-Assay Analysis

**Figure 4:**
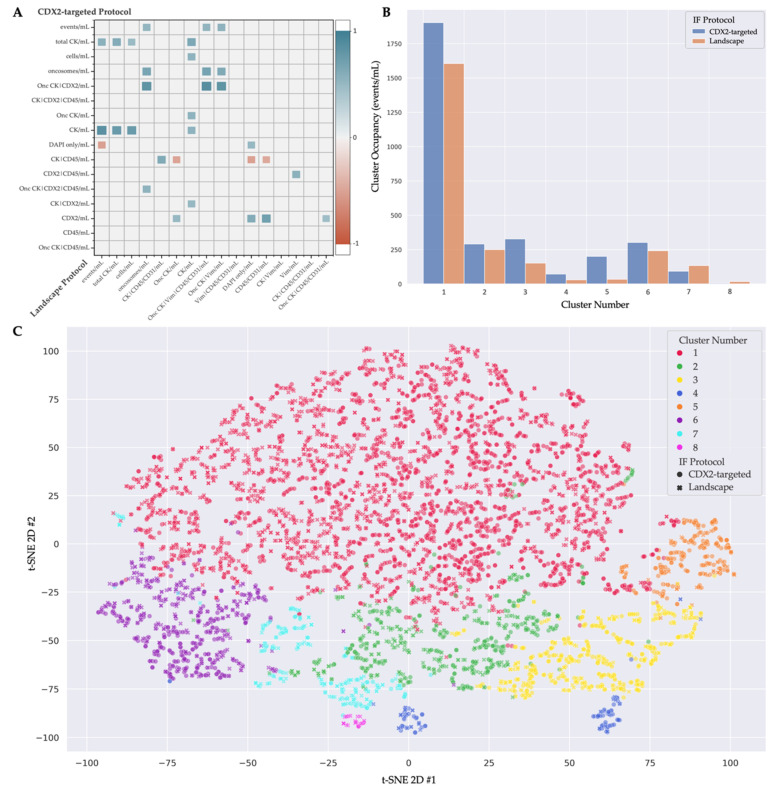

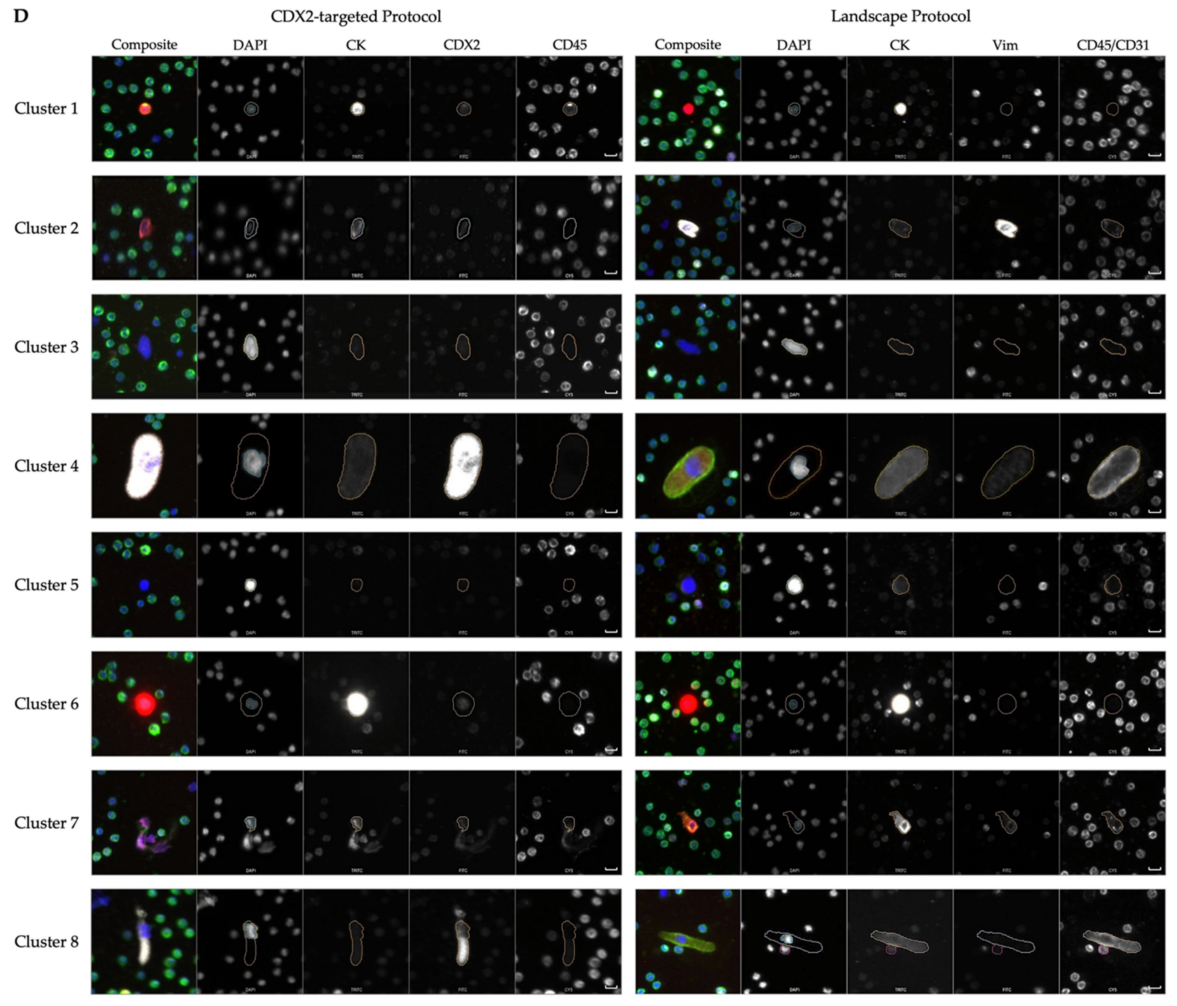
Multi-assay analysis of the Landscape and CDX2-targeted immunofluorescence (IF) assays analyzed by OCULAR. (**A**) Statistically significant (*p* ≤ 0.05) rare-event-count correlations across the two IF assays, with the red-to-blue color gradient indicating a negative-to-positive correlation, respectively. (**B**) Cluster occupancy of the rare cells identified by the CDX2-targeted and Landscape stains when using the 8-group hierarchical clustering model. (**C**) t-Stochastic neighbor embedding (t-SNE) plot of the 8 cell clusters comprised of rare cells from both stains, as indicated by the markers’ shape. (**D**) Representative gallery of the 8 clusters with cells from both assays. Each row represents a cluster with a cell from the CDX2-targeted protocol on the left and a cell from the Landscape protocol on the right. Images taken at 100× magnification. Scale bars represent 100 µm.

A multi-assay comparison was conducted on samples stained both with the CDX2-targeted and Landscape IF protocols. An analysis of the rare-event enumerations from both staining protocols with matched samples revealed various positive and negative correlations between channel-type classification counts (Figure 4A). Specific rare cell types detected by both IF protocols were positively correlated, such as the DAPI-only cells (*p* = 0.032, *τ* = 0.51) and the CK-only cells (*p* = 0.018, *τ* = 0.55). Similar positive correlations were found among the aggregate classifications, including the oncosomes (*p* = 0.003, *τ* = 0.65) and total CK-positive rare-event population (*p* = 0.006, *τ* = 0.62). A positive association between rare events that differ by one unshared biomarker between the stains potentially indicates a single rare-event type. An example of this is the CK-only cell counts from the Landscape-staining protocol being positively associated with the CK|CDX2 cells from the CDX2 IF protocol (*p* = 0.029, *τ* = 0.51). A similar pattern was observed within the oncosome population, with the Onc CK|Vim being positively correlated to the Onc CK|CDX2 (*p* < 0.001, *τ* = 0.82). The significant negative correlations across the assays included the DAPI-only cells from Landscape-staining protocol and the CK|CD45 cells from the CDX2 IF protocol (*p* = 0.029, *τ* = −0.51). Interestingly, if selected for rarity, the CD45/CD31 population from the Landscape-staining protocol was uncorrelated with the CD45 population from the CDX2-staining protocol (*p* = 0.645, *τ* = 0.12), indicating that the differential IF expression was likely due to the addition of the CD31 biomarker.

OCULAR presents morphometrics related to the size and shape of the cell and nucleus. A clustering analysis of the morphometric features was conducted to characterize the rare-event types across the two IF staining protocols, improving our understanding of the analytes in liquid biopsy. Hierarchical clustering into eight groups afforded the most discrete separation of the hypothesized megakaryocytes and endothelial cells. It is important to note that these highlighted rare cell types are not entirely separated into their own clusters, indicating the morphological heterogeneity within the cell categories. The distribution of cells from both IF staining protocols into the eight clusters and representative images are provided in Figure 4B–D. Table 2 provides a description of each cluster.

### 3.4. Clinical Correlation of Liquid-Biopsy Data

**Figure 5:**
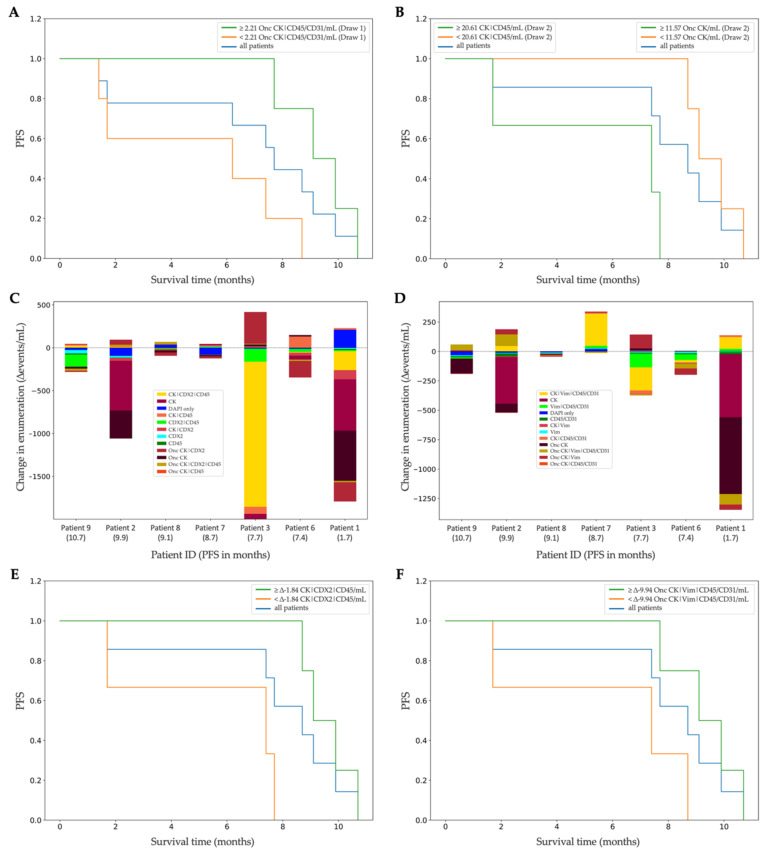
Survival analysis of rare events from the Landscape- and CDX2-targeted immunofluorescence protocols. (**A**) Kaplan-Meier (KM) curve showing that patients with more than the median of 2.21 Onc CK|CD45/CD31/mL found in Draw 1 by the Landscape protocol had longer progression-free survival (PFS). (**B**) KM curve showing two rare events with survival implications found in Draw 2 by the CDX2-targeted protocol. Patients with more than the median of 20.61 CK|CD45 cells/mL or more than the median of 11.57 Onc CK/mL had shorter PFS. (**C**) Rare-event kinetics between Draw 1 and Draw 2 analyzed with the CDX2-targeted protocol, ordered from longest to shortest PFS. (**D**) Rare-event kinetics between Draw 1 and Draw 2 analyzed with the Landscape protocol, ordered from longest to shortest PF€(**E**) KM curve showing that patients with a change of ≥−1.84 CK|CDX2|CD45 cells/mL from the CDX2-targeted protocol had longer PFS. (**F**) KM curve showing that patients with a change of ≥−9.94 Onc CK|Vim|CD45/CD31/mL from the Landscape protocol had longer PFS.

Next, we investigated the clinical relevance of the rare events detected in the liquid biopsy. PFS was reported for nine of the ten patients (mean PFS = 6.98 months). A survival analysis of the rare events identified by the Landscape assay in Draw 1 samples revealed a positive correlation between the number of Onc CK|CD45/CD31 and PFS (*p* = 0.0372, *τ* = 0.70), as patients with ≥2.21 oncosomes/mL had an improved survival (Figure 5A). A similar analysis of the populations identified by the CDX2-targeted assay in Draw 2 samples revealed two rare-event types negatively associated with PFS: CK|CD45 cells (*p* = 0.0362, *τ* = −0.77) and Onc CK (*p* = 0.0068, *τ* = −0.89). Patients with ≥20.61 CK|CD45 cells/mL or ≥11.57 Onc CK/mL had poor survival (Figure 5B).

Beyond timepoint-static enumerations, the rare-event kinetics (Figure 5C,D), defined as the change in rare-event subtypes between draws, were significantly associated with PFS. Of the nine patients with survival data, two were missing Draw 2; therefore, only seven patients were included in the kinetics survival analysis. The changes in the CK|CDX2|CD45 cells (*p* = 0.0068, *τ* = 0.89) and the Onc CK|Vim|CD45/CD31 (*p* = 0.0025, *τ* = 0.93) were positively associated with PFS (Figure 5E,F).

## 4. Discussion

As a minimally invasive approach, the liquid biopsy has the potential to significantly advance patient care by addressing current clinical challenges in mCRC. In this study, we show a comprehensive profile of the liquid biopsy that encapsulates the heterogeneous CTC and oncosome populations while providing the initial clinical validation of the liquid biopsy in mCRC patients.

This study corroborates findings of a limited CK-only CTC population in mCRC [25,26,27]. mCRC is known for its extensive solid tumor heterogeneity, and herein, we show interpatient and intrapatient heterogeneity in the circulating rare-event population. The Landscape protocol aided in the phenotypic characterization of morphologically distinct cell types, allowing for the detection of new rare-cell populations, such as circulating endothelial cells (CECs). Initially identified in the 1970s [60], CEC counts have been shown to be elevated in the liquid biopsy of cancer patients [61]. In mCRC, tumor-derived CECs have been used as a prognostic indicator of clinical response to first-line therapies [62] and patient survival [41,42]. Interestingly, a previous investigation targeting CTCs in CRC instead discovered tumor-derived CEC clusters that starkly differentiated normal donors, treatment-naïve and early-stage patients [63]. The detection of clinically significant CECs while attempting to find CTCs highlights the importance of using an unbiased rare-cell approach to capturing the heterogeneity in the liquid biopsy of mCRC. In this study, the morphologically distinct Vim|CD45/CD31 cells with variable CK expression were found individually and in clusters. Based on their morphology and biomarker expression, we expect that these cells are CECs. While downstream proteomics and genomics are needed to confirm this cell lineage, the filamentous Vim and punctate CD45/CD31 signal captured by the Landscape IF protocol are characteristic of endothelial cells [64,65]. HDSCA has previously identified CECs in the liquid biopsy, the likes of which present a similar morphology and biomarker expression pattern [33].

We additionally identified large, morphologically distinct CD45/CD31 cells in the mCRC samples that appear to be multilobular with sizeable nuclei and punctate cytoplasmic expression. Such cellular features lead us to hypothesize that these events are megakaryocytes. These platelet-producing cells derived from the bone marrow are positive in CD31, have a granular cytoplasm and are large, with up to a 160 µm diameter [66]. Found either in the solid tumor microenvironment or in circulation, megakaryocytes have shown to have prognostic potential in prostate [67] and non-small-cell lung cancer [68]. While platelet indices have served as diagnostic biomarkers in mCRC [69], the novel identification of potential megakaryocytes in this cancer type could have significant clinical implications even in the absence of direct platelet detection. Future studies should characterize these megakaryocyte candidates with downstream proteomics to confirm their lineage and to compare their enumerations to patient-specific clinical factors and survival. This unique cell type further exemplifies the heterogeneity of the circulating cell profile of mCRC and highlights the power of rare-event detection systems to represent it.

This study demonstrates that the utility of the liquid biopsy is not limited to cellular events, emphasizing the importance of detecting vesicles. Vesicles have been described and classified according to the mechanism of cellular release and size, which may be dependent on the method of detection or isolation [44,70]. Oncosomes comprised a significant portion of the rare-event profiles from both the CDX2-targeted and Landscape assays in mCRC patient samples. HDSCA has previously identified oncosomes with similar size and biomarker expression in prostate cancer [46], bladder cancer [48] and upper tract urothelial carcinoma [51], suggesting that these events may be found in a variety of cancer types. Most importantly, oncosome enumerations from both IF protocols correlated with PFS in this cohort. The kinetics of a Landscape-stained oncosome population also correlated significantly with PFS, pointing to the importance of enumerating these analytes over time. The potential for making diagnoses, prognoses and subsequent treatment decisions based off oncosomes, especially in a cancer type like mCRC that does not widely present CK-only CTCs, warrants further studies to molecularly characterize these events. Herein, we show that oncosomes associated with mCRC tumorigenesis may be useful prognostic biomarkers.

Predicated on this study’s sample-matched design, the multi-assay analysis is a novel attempt to overlap IF protocols using the shared features of an unbiased rare-event detection platform. The successful grouping of known cell types into delineated clusters, such as the Epi.CTCs in cluster 6, serves as a proof of concept for applications in a larger cohort. Using only image-based morphometrics, the multi-assay analysis increases the number of evaluable IF biomarkers for a single sample, while maintaining a low monetary cost relative to single-cell proteomics. Cluster 4 best depicts the value of additional biomarkers in understanding the cellular biology. Using the Landscape assay, we detected megakaryocytes with a CD45/CD31-positive signal, while in the CDX2-targeted assay, we detected megakaryocyte-like cells that presented a CDX2-positive but CD45-negative signal. The shared feature analysis allowed for the identification of these cells as similar and the observation of CDX2 antibody binding to these cells. A multi-assay approach is uniquely suited for rare-event analysis, wherein thresholds from multiple IF protocols conducted in tandem could demonstrate the clinical utility of the liquid biopsy.

Phenotypic switches are fundamental to CRC initiation, metastasis and relapse [71,72,73], thus requiring longitudinal prognostic tools and changing therapies to target the continuously evolving cell types. The minimally invasive liquid biopsy has the potential to address this challenge, and we show initial evidence for clinical utility. The molecular characterization of the rare events detected in this study will elucidate their potential role in mCRC tumorigenesis. The HDSCA3.0 workflow includes the capability for genomic analysis, both SNV (single-nucleotide variation) and CNV (copy-number variation), for both single cells and cell-free DNA (cfDNA) [74,75,76], as well as targeted multiplexed proteomic analysis [38,75] on samples previously characterized at the morphological and phenotypic level by IF. The data presented here serves to motivate further genomic and/or proteomic analysis of the CTCs and oncosomes detected to validate their neoplastic origin and association with the disease state.

Additional studies are needed with a greater patient sample size, PB draws from multiple timepoints throughout treatment and patient-specific clinical information (KRAS/NRAS status, TNM staging, etc.) to provide the prognostic, diagnostic and predictive utility of the liquid biopsy in the management of mCRC.

## 5. Conclusions

As one of the world’s most prevalent oncological diseases, mCRC poses numerous clinical challenges due to its extensive tumor heterogeneity. This study establishes evidence for the clinical validation of an unbiased rare-event approach to the liquid biopsy. For the first time, we demonstrate the value of a comprehensive CTC and oncosome detection approach in the PB of mCRC. Our results highlight the heterogeneity of the liquid-biopsy profile with the identification of rare-event frequencies unique to mCRC patients. By analyzing both IF expression patterns and morphological parameters, we identify two select rare cell types that warrant future study into their implications in mCRC. Furthermore, we demonstrate the utility of analyzing multiple IF assays in tandem to characterize the heterogeneous populations detected. These findings motivate the further molecular characterization of these analytes and investigation into their predictive power with respect to patient outcomes in mCRC.

## Figures and Tables

**Table 1:** Rare-event frequencies, enumerations and sample positivity from the Landscape and CDX2-targeted immunofluorescence (IF) protocols. The sample positivity threshold of ≥5 events/mL was determined by comparisons to the rare-event enumerations of a randomly selected normal donor cohort. The frequency of each classification is provided as a percentage of the total rare-event profile for each IF protocol.

IF	Event Classification	Sample Positivity	Mean (Events/mL)	Standard Error (±Events/mL)	% of Total Rare Events	Median (Events/mL)	Range (Events/mL)
Protocol							
Landscape	DAPI only	15/18	13.69	2.9	3.55	10.84	0.00–51.57
	CK (Epi.CTC)	18-Jul	57	36.36	14.77	3.55	0.00–549.63
	Vim	18-Nov	6.64	1.27	1.72	7.3	0.00–20.16
	CD45/CD31	18-Oct	10.29	2.44	2.67	8.05	0.00–33.16
	CK|Vim (Mes.CTC)	18-Jun	7.6	1.4	1.97	1.4	0.00–91.29
	CK|CD45/CD31	18-Apr	4.66	1.86	1.21	2.13	0.00–30.74
	Vim|CD45/CD31	18-Nov	24.52	8.73	6.35	7.79	0.00–121.00
	CK|Vim|CD45/CD31	15/18	100.4	35.02	26.01	12.89	0.00–453.13
	Onc CK	18-Dec	71.59	38.39	18.55	6.64	1.06–657.60
	Onc CK|Vim	18-Oct	32.72	13.64	8.48	7.38	0.00–217.65
	Onc CK|CD45/CD31	0/18	1.47	0.36	0.38	1.12	0.00–4.47
	Onc CK|Vim|CD45/CD31	14/18	55.37	16.22	14.35	34.32	0.00–268.32
CDX2- targeted	DAPI only	18/18	63.72	12.8	8.33	45.29	16.94–226.94
	CK	18-Dec	88.14	43.95	11.53	11.97	0.00–597.32
	CDX2	18-Dec	11.45	3.28	1.5	7.27	1.04–60.22
	CD45	18-Mar	5.44	3.35	0.71	0	0.00–59.22
	CK|CDX2 (CDX2.CTC)	14/18	19.95	6.71	2.61	11.34	0.00–124.08
	CK|CD45	18-Dec	36.66	13.71	4.79	10.02	0.00–203.63
	CDX2|CD45	18-Dec	29.37	12.46	3.84	9.8	0.00–185.68
	CK|CDX2|CD45	14/18	143.67	101.35	18.79	21.79	1.44–1843.34
	Onc CK	15/18	123.55	60.73	16.16	26	2.31–1035.65
	Onc CK|CDX2	18/18	222.4	65.04	29.08	114.87	15.55–1151.64
	Onc CK|CD45	0/18	0.06	0.06	0.01	0	0.00–1.06
	Onc CK|CDX2|CD45	18-Oct	20.27	7.64	2.65	14.5	0.00–1138.28

**Table 2:** Cellular description for the multi-assay cluster analysis. Cluster occupancy of the rare cells identified by the CDX2-targeted and Landscape staining protocols when using the 8-group hierarchical clustering model for multi-assay analysis.

Cluster Number	Cluster Comments	Cells from Landscape	Comments from Landscape	Cells from CDX2-Targeted	Comments from CDX2-Targeted
1	Heterogeneous phenotype with cellular morphology similar to WBCs	1605	Most prominent: CK|Vim|CD45/CD31	1903	Most prominent: CK|CDX2|CD45
2	Includes endothelial cells	249	Morphologically distinct Vim|CD45/CD31 cells with variable CK expression	291	64 (22%) DAPI-only, 67 (23%) CK and 34 (12%) CK|CDX2 cells
3	Large nuclei, more eccentric than cluster 5	152	101 (66%) DAPI only, 11 (7%) morphologically distinct CD45/CD31 expressing cells	328	279 (85%) DAPI-only
4	Includes megakaryocytes	30	25 (83%) morphologically distinct CD45-/CD31-expressing cells, 5 (17%) small rod-like CD45-/CD31-expressing cells	72	DAPI-only and CDX2-only with similar large morphology
5	Large nuclei, more circular than cluster 3	34	15 (44%) DAPI-only	201	191 (95%) DAPI-only
6	CK only CTCs	242	219 (90%) Epi.CTCs	302	265 (88%) CK and 29 (10%) CK|CDX2 cells
7	Includes endothelial cells	134	84 (63%) morphologically distinct Vim|CD45/CD31 cells with variable CK expression	93	80 (86%) CK only. Morphologically distinct from cluster 6, more elongated.
8	Includes megakaryocytes	18	Morphologically distinct CD45/CD31 cells with variable CK and Vim expression	6	Large, morphologically distinct cells with punctate CDX2 expression

## Data Availability

All data discussed in this manuscript are included in the main manuscript text or Supplementary Materials. Some of the data can be accessed through our website http://pivot.usc.edu/. The imaging data is available through the BloodPAC Data Commons Accession ID “BPDC000124” (https://data.bloodpac.org/discovery/BPDC000124).

## Supplementary Materials

The following supporting information can be downloaded at: https://www.mdpi.com/article/10.3390/cancers14194891/s1, Figure S1. Enumeration of the rare events detected in the Landscape-stained ND samples analyzed by OCULAR. Table S1. Rare-event frequencies, enumerations, and sample positivity from the enrichment-based and OCULAR analysis of the samples stained with the CDX2-targeted assay. The sample positivity threshold of ≥5 events/mL was determined by comparisons to the rare-event enumerations of an ND cohort. Within each detection approach, the frequency of each classification is provided as a percentage of the total rare-event profile. Figure S2. CK-positive rare events from the CDX2-stained samples analyzed by OCULAR. (A) Cells positioned on the edge of frames that are intentionally removed by the quality-control process in OCULAR, representative of the 2.04% non-concordance group between the two detection approaches. Frame edges are indicated by the dashed white lines. (B) CK-positive cells, containing representative HD-CTC, CTC-Apoptotic and two CTC-Small candidates as per the CK-focused approach. (C) CK|CDX2 cells, containing a CTC cluster, cells with a dim CK signal, and eccentric rare cells. (D) CK|CD45 cells with varying CD45 expression. (E) CK|CDX2|CD45 cells. (F) Heterogenous CK positive oncosomes. DAPI: blue, CK: red, CDX2: white, CD45: green. Images taken at 100× magnification. Scale bars represent 10 µm. Figure S3. CDX2-stained samples rare-event enumeration using HDSCA1.0. (A) Enumeration (CTCs/ml) of each CTC subtype using the CTC-focused approach per patient and draw. (B) Frequency (%) of each CTC subtype using the CTC-focused approach per patient and draw.
