# Supplementary material for: Defining A Liquid Biopsy Profile of Circulating Tumor Cells and Oncosomes in Metastatic Colorectal Cancer for Clinical Utility
Source: Cancers (Basel). 2022 Oct 6;14(19):4891. doi: 10.3390/cancers14194891 (PMC9563925; doi:10.3390/cancers14194891)
Supplement: Supplementary file 1 [file cancers-14-04891-s001.zip › cancers-1936115-supplementary.pdf]

Supplemental Information

CK-focused Approach

The High Definition Single Cell Assay (HDSCA) first generation employed a rare cell detection approach focused on cytokeratin (CK) only circulating tumor cells (CTCs). Image data sets were analyzed for the presence of CK positive and CD45 negative cells. Cells were classified as HD-CTCs using the following criteria: CK-positive, CD45-negative, morphologically distinct from surrounding white blood cells (WBCs) and contained an intact DAPI nucleus without observable apoptotic changes or a disrupted appearance. This cellular classification represents the field's standard definition of a CTC. Marginal populations included: 1) CTC-Small: CK-positive, CD45-negative cells with intact nuclei that were the same size or smaller than neighboring WBCs; 2) CTC-NoCK: cells with CK levels lower than HD-CTCs or absent, CD45-negative, and large morphological distinct nuclei, and 3) CTC-Apoptotic: CK-positive, CD45-negative cells with a DAPI pattern of nuclear condensation and fragmentation and plasma membrane blebs that are common features of apoptotic cells. Cell classifications were made and confirmed by multiple hematopathologist-trained technical analysts.

Equivalent CTC Detection

We further utilized the imaging data set from the CDX2 immunofluorescence (IF) protocol to understand the differences in the cellular detection approaches (CK-focused or OCULAR) through a comparative analysis of liquid biopsy technologies in their ability to represent the circulating rare cell profile of a patient. The CDX2-stained samples were analyzed by the CK-focused approach and OCULAR, to determine the utility of an unbiased algorithm for event detection.

To compare the two cellular identification algorithms, OCULAR and the CK-focused approach were evaluated for the detection of CTCs. The OCULAR methodology found 97.96% of the CTC candidates identified by a CK-focused approach. Furthermore, the 2.04% non-concordance between the two algorithms was the result of additional quality control steps in OCULAR (Figure S2A). Moving beyond the CK-positive events, OCULAR found significantly more CK negative rare cells ($p < 0.0001$) than the CK-focused approach. On average, for each sample stained by the CDX2-targeted protocol, OCULAR increased the rare event population of interest by tenfold (10.18x) from the CK-focused approach (Figure S3). OCULAR's effective detection of CK-positive cells and a broader rare event population that was missed in a CK-focused analysis supports the utility of an unbiased approach.

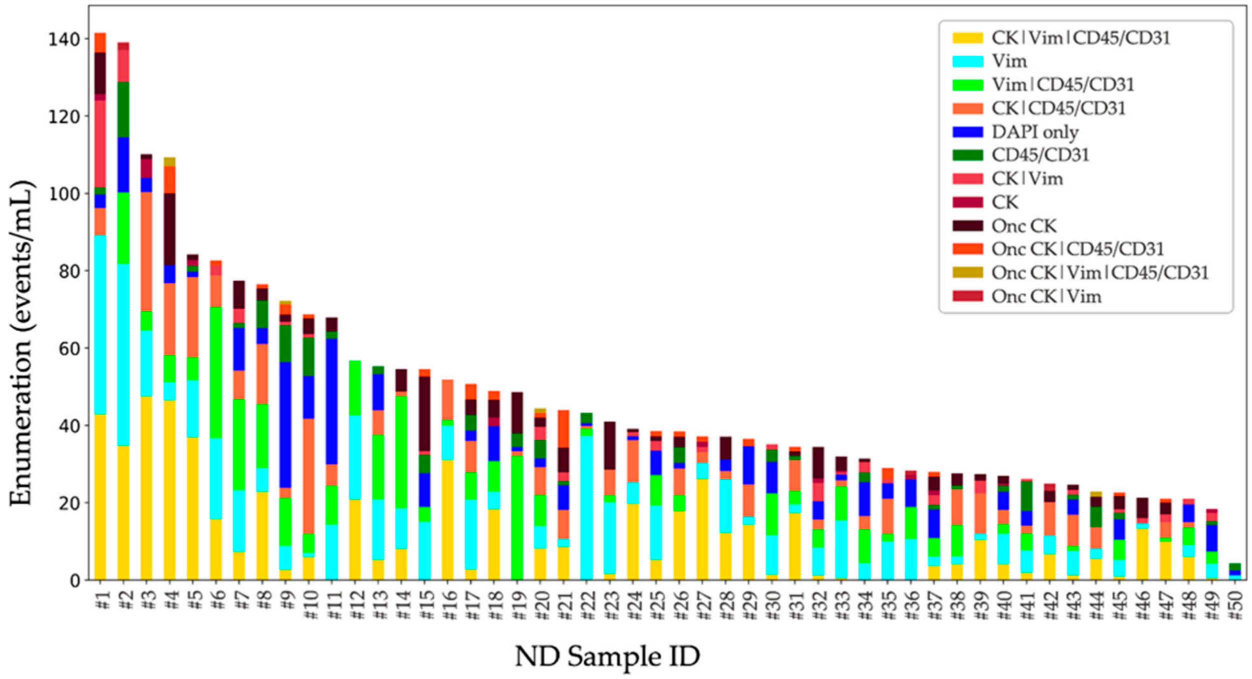

Figure S1. Enumeration of the rare events detected in the Landscape-stained normal donor samples analyzed by OCULAR.

Table S1. Rare event frequencies, enumerations, and sample positivity from the enrichment-based and OCULAR analysis of the samples stained with the CDX2-targeted assay. The sample positivity threshold of ≥5 events/ml was determined by comparisons to rare event enumerations of a normal donor cohort. Within each detection approach, the frequency of each classification is provided as a percentage of the total rare event profile.

Detection Approach	Event Classification	Sample Positivity	Mean (events/ml)	Standard Error (± events/ml)	% of total rare events	Median (events/ml)	Range (events/ml)
CK-focused	HD-CTC	7/18	45.88	24.33	58.90	3.52	0.00-354.01
	CTC-NoCK	14/18	17.50	5.07	22.47	11.54	0.99-64.06
	CTC-Apoptotic	2/18	7.75	5.78	9.95	0.39	0.00-103.10
	CTC-Small	2/18	6.76	4.29	8.68	0.00	0.00-65.07
OCULAR	DAPI only	18/18	63.72	12.80	8.03	45.29	16.94-226.94
	CK	12/18	88.14	43.95	11.11	11.97	0.00-597.32
	CDX2	12/18	11.45	3.28	1.44	7.27	1.04-60.22
	CD45	3/18	5.44	3.35	0.69	0.00	0.00-59.22
	CK I CDX2	14/18	19.95	6.71	2.52	11.34	0.00-124.08
	CK I CD45	12/18	36.66	13.71	4.62	10.02	0.00-203.63
	CDX2 I CD45	12/18	29.37	12.46	3.70	9.80	0.00-185.68
	CK I CDX2 I CD45	14/18	143.67	101.35	18.11	21.79	1.44-1843.34
	Onc CK	15/18	123.55	60.73	15.58	26.00	2.31-1035.65
	Onc CDX2	15/18	28.27	7.17	3.56	17.32	0.00-104.45
	Onc CK I CDX2	18/18	222.40	65.04	28.04	114.87	15.55-1151.64
	Onc CK I CD45	0/18	0.06	0.06	0.01	0.00	0.00-1.06
	Onc CDX2 I CD45	0/18	0.16	0.16	0.02	0.00	0.00-2.96
	Onc CK I CDX2 I CD45	10/18	20.27	7.64	2.56	14.50	0.00-1138.28

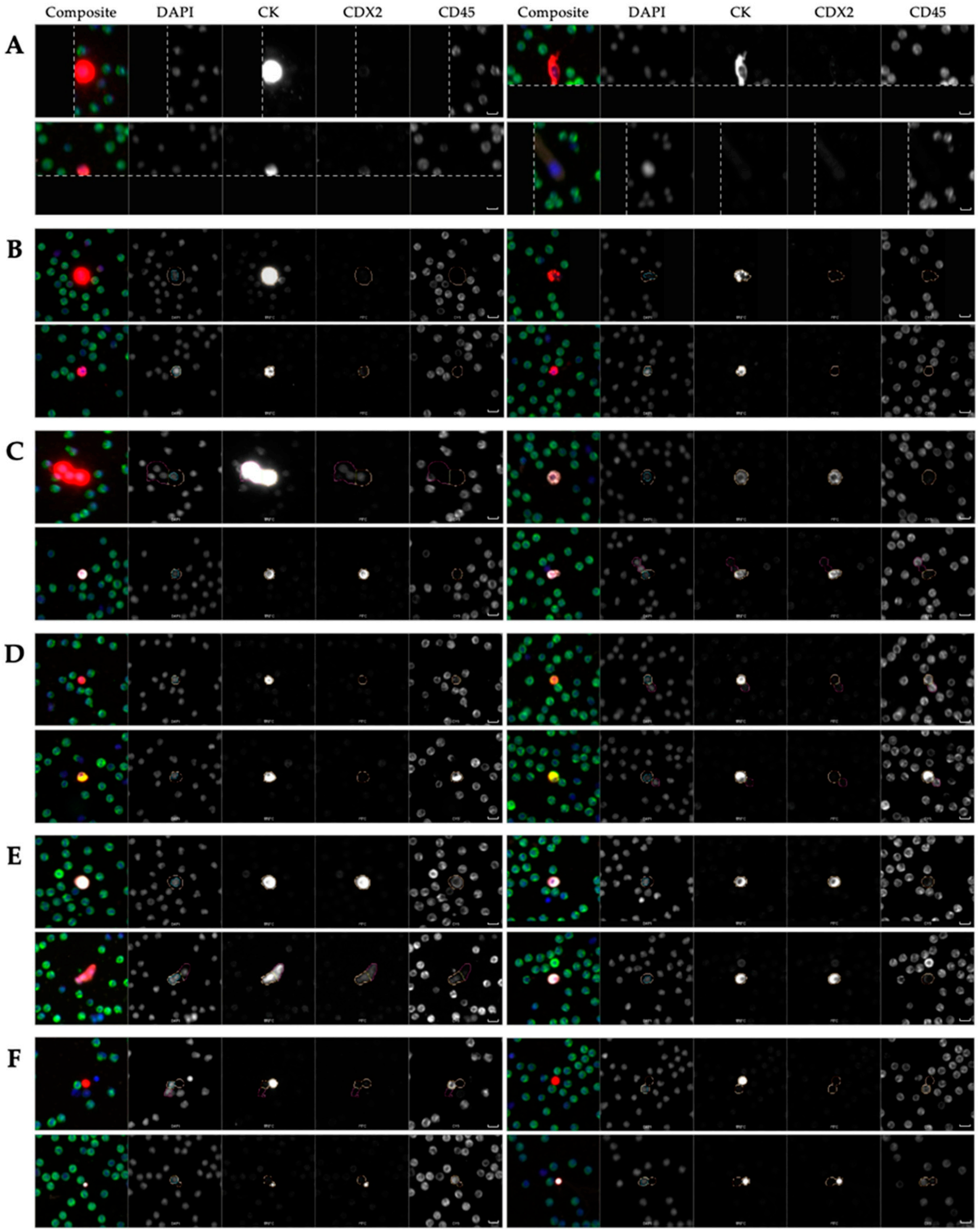

Figure S2. CK-positive rare events from the CDX2-stained samples analyzed by OCULAR. (A) Cells positioned on the edge of frames that are intentionally removed by the quality control process in OCULAR, representative of the 2.04% non-concordance group between the two detection approaches. Frame edges are indicated by the dashed white lines. (B) Cytokeratin (CK) positive cells, containing representative HD-CTC, CTC-Apoptotic, and two CTC-Small candidates as per the CK-focused approach. (C) CK|CDX2 cells, containing a circulating tumor cell cluster, cell with dim CK signal, and eccentric rare cell. (D) CK|CD45 cells with varying CD45 expression. (E) CK|CDX2|CD45 cells. (F) Heterogenous CK positive oncosomes. DAPI: blue, CK: red, CDX2: white, CD45: green. Images taken at 100× magnification. Scale bars represent 10 μm.

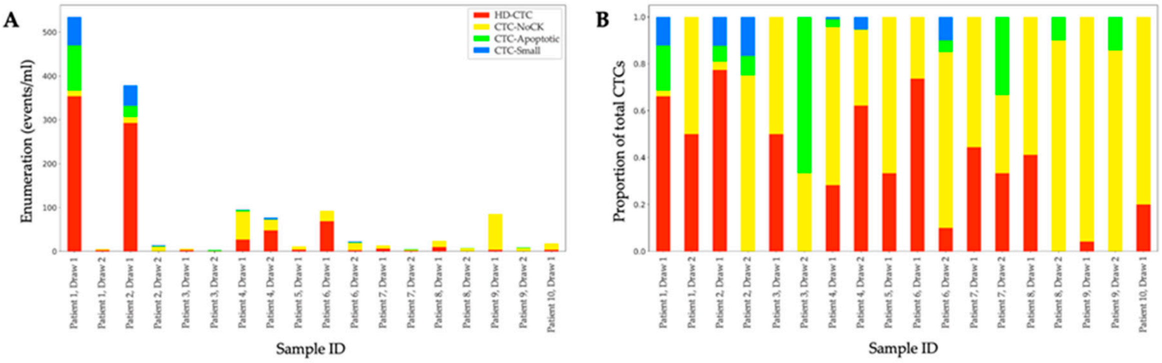

Figure S3. CDX2-stained samples rare event enumeration using HDSCA1.0. (A) Enumeration (CTCs/ml) of each circulating tumor cell (CTC) subtype using the CK-focused approach per patient and draw. (B) Frequency (%) of each CTC subtype using the CK-focused approach per patient and draw.
